# Association of increased extracellular volume expansion with elevated left ventricular end diastolic pressure in patients with nonischemic cardiomyopathy

**DOI:** 10.1186/1532-429X-16-S1-P316

**Published:** 2014-01-16

**Authors:** Jie J Cao, Lynette J Duncanson, Michael Passick, Joshua Y Cheng, Kathy Halloran

**Affiliations:** 1 St Francis Hospital, Roslyn, New York, USA; 2 Cardiology, State University of New York at Stony Brook, Stony Brook, New York, USA

## Background

Myocardial extracellular volume expansion is a marker of myocardial fibrosis which is associated with myocardial stiffness and impaired relaxation property. In this study we investigated the association of extracellular volume indices using T1 mapping and left ventricular end diastolic pressure (LVEDP) using left atrial transit time in patients with nonischemic cardiomyopathy.

## Methods

All subjects were prospectively recruited to undergo cardiac MRI in a 1.5 T scanner. Modified Look-Locker Inversion recovery sequence was used with motion correction included in the post processing algorithm. Pre-contrast and post contrast (20 minutes after gadolinium injection at 0.15 mmol/kg) T1 maps of the blood pool and myocardium were assessed in basal, mid and apical segments of the short axis planes using Siemens software. Calculated T1 indices were the average value of the basal and mid slice which included the pre- and post-contrast myocardial T1, contrast partition coefficient using the ratio of signal change in blood and myocardium before and after contrast administration, and extracellular volume fraction (ECV) which was partition coefficient multiplied with one minus hematocrit to account for the blood contrast volume of distribution. LVEDP was assessed using normalized mean left atrial transit time derived from the time-signal intensity curve of the first pass perfusion image during gadolinium injection at 0.01 mmol/kg.

## Results

Compared to the normal controls (N = 8) patients with nonischemic cardiomyopathy (N = 31) had lower LV ejection fraction (EF) (47 ± 12% vs 54 ± 3%, p = 0.021) and higher LVEDP (14 ± 5 mmHg vs 10 ± 2 mmHg, p = 0.014). Using Pearson's correlation increased partition coefficient, ECV and pre-contrast myocardial T1 were significantly correlated with elevated LVEDP: r = 0.385 (p = 0.021), r = 0.355 (p = 0.029) and r = 0.335 (p = 0.04), respectively. In dichotomized analysis elevated LVEDP (≥13 mmHg, N = 13) was associated with significantly higher partition coefficient (0.51 ± 0.1 vs 0.45 ± 0.06, p = 0.004), higher ECV (0.30 ± 0.06 vs 0.26 ± 0.03, p = 0.002) and higher pre-contrast myocardial T1 (1040 ± 123 ms vs 983 ± 44 ms, p = 0.045) when compared to those with normal LVEDP ( < 13 mmHg, N = 26). Among patients with elevated LVEDP (average 19 ± 3 mmHg) there were no significant differences in partition coefficient, ECV and pre-contrast myocardial T1 between those with preserved or reduced LVEF. In contrast, there was a lack of significant association between post-contrast myocardial T1 and LVEDP.

## Conclusions

Increased contrast partition coefficient, ECV and pre-contrast myocardial T1 were significantly associated with elevated LVEDP in patients with nonischemic cardiomyopathy thereby supporting the link between extracellular volume expansion and myocardial diastolic dysfunction. Quantitative tissue characterization combined with hemodynamic assessment underscores the value of cardiac MRI in the evaluation of nonischemic cardiomyopathy.

## Funding

St Francis Research Fundation.

